# Childhood Langerhans cell histiocytosis with severe lung involvement: a nationwide cohort study

**DOI:** 10.1186/s13023-020-01495-5

**Published:** 2020-09-09

**Authors:** Solenne Le Louet, Mohamed-Aziz Barkaoui, Jean Miron, Claire Galambrun, Nathalie Aladjidi, Pascal Chastagner, Kamila Kebaili, Corinne Armari-Alla, Anne Lambilliotte, Julien Lejeune, Despina Moshous, Valeria Della Valle, Chiara Sileo, Hubert Ducou Le Pointe, Jean-François Chateil, Sylvain Renolleau, Jean-Eudes Piloquet, Aurelie Portefaix, Ralph Epaud, Raphaël Chiron, Emmanuelle Bugnet, Gwenaël Lorillon, Abdelatif Tazi, Jean-François Emile, Jean Donadieu, Sébastien Héritier

**Affiliations:** 1 French Reference Center for Langerhans Cell Histiocytosis, Trousseau Hospital, 26 avenue du Dr Netter, 75012 Paris, France; 2 Department of Pediatric Hematology and Oncology, Hôpital de la Timone, Marseille, France; 3 Department of Pediatric Hematology and Oncology, Centre Hospitalo-Universitaire de Bordeaux, Bordeaux, France; 4 Department of Pediatric Hematology and Oncology, Brabois-Enfants Hospital, Centre Hospitalo-Universitaire de Nancy, Vandœuvre-lès-Nancy, France; 5 Department of Paediatric Oncology, Institut d’Hémato-Oncologie Pediatrique, Lyon, France; 6 Department of Pediatric Hematology and Oncology, Centre Hospitalo-Universitaire de Grenoble, La Tronche, France; 7 Department of Pediatric Hematology and Oncology, Centre Hospitalo-Universitaire de Lille, Lille, France; 8 Department of Pediatric Hematology and Oncology, Centre Hospitalo-Universitaire de Tours, Tours, France; 9 Department of Pediatric Immunology, Hematology and Rheumatology, Necker Hospital, Assistance Publique–Hôpitaux de Paris, Paris, France; 10 Institut Imagine, Sorbonne University, Paris, France; 11 Department of Radiology, Trousseau Hospital, Assistance Publique–Hôpitaux de Paris, Paris, France; 12 Department of Radiology, Centre Hospitalo-Universitaire de Bordeaux, Bordeaux, France; 13 Intensive care unit, Necker Hospital, Assistance Publique–Hôpitaux de Paris, Paris, France; 14 Intensive care unit, Trousseau Hospital, Assistance Publique–Hôpitaux de Paris, Paris, France; 15 Intensive care unit, Lyon Hospices Civils, Lyon, France; 16 Service de Pédiatrie générale, CHIC, Créteil, France; 17 Service de Pneumologie, Arnaud de Villeneuve Hospital, Montpellier, France; 18 Service de Pneumologie Centre de référence des histiocytoses Hôpital Saint Louis, Paris, France; 19 Paris University, INSERM U976, Paris, France; 20 EA4340, UVSQ, Paris-Saclay University, Boulogne-Billancourt, France; 21 Départment of Pediatric Hematology and Oncology, Sorbonne University, Paris, France

**Keywords:** Childhood, Pulmonary, Langerhans cell histiocytosis, Targeted therapy, Intensive care, Severe

## Abstract

**Background:**

Lung involvement in childhood Langerhans cell histiocytosis (LCH) is infrequent and rarely life threatening, but occasionally, severe presentations are observed.

**Methods:**

Among 1482 children (< 15 years) registered in the French LCH registry (1994–2018), 111 (7.4%) had lung involvement. This retrospective study included data for 17 (1.1%) patients that required one or more intensive care unit (ICU) admissions for respiratory failure.

**Results:**

The median age was 1.3 years at the first ICU hospitalization. Of the 17 patients, 14 presented with lung involvement at the LCH diagnosis, and 7 patients (41%) had concomitant involvement of risk-organ (hematologic, spleen, or liver). Thirty-five ICU hospitalizations were analysed. Among these, 22 (63%) were secondary to a pneumothorax, 5 (14%) were associated with important cystic lesions without pneumothorax, and 8 (23%) included a diffuse micronodular lung infiltration in the context of multisystem disease.

First-line vinblastine–corticosteroid combination therapy was administered to 16 patients; 12 patients required a second-line therapy (cladribine: *n* = 7; etoposide-aracytine: *n* = 3; targeted therapy *n* = 2). A total of 6 children (35%) died (repeated pneumothorax: *n* = 3; diffuse micronodular lung infiltration in the context of multisystem disease: *n* = 2; following lung transplantation: *n* = 1). For survivors, the median follow-up after ICU was 11.2 years. Among these, 9 patients remain asymptomatic despite abnormal chest imaging.

**Conclusions:**

Severe lung involvement is unusual in childhood LCH, but it is associated with high mortality. Treatment guidelines should be improved for this group of patients: viral infection prophylaxis and early administration of a new LCH therapy, such as targeted therapy.

## Background

Langerhans cell histiocytosis (LCH) is a rare disease that occurs more frequently in children than in adults. LCH has extremely variable clinical manifestations [1, 2]. It is a clonal disorder characterized by an accumulation of pathological CD1a + CD207+ histiocytes, affecting nearly all organ systems, and thus, leading to a myriad of clinical manifestations. The lungs are involved in approximately 10% of children with LCH. Somatic mutations are typically found in a proto-oncogene in the MAPKinase pathway (mostly *BRAF*^V600E^) [3, 4]. For each patient, the extension of the disease is established based on the classification of the international medical society of histiocytic disorders (Histiocyte Society). This classification considers affected organs number, lung involvement and the risk of organ involvement (RO+), defined as the liver, spleen, and haematological system. Currently, the lung is no longer considered an RO [5–7]. Pulmonary involvement in LCH (PLCH), is typically observed in two distinct clinical presentations in children [2, 7, 8]. In one presentation, lung involvement in young children is mostly associated with a multisystemic disease (MS) with RO involvement. It remains in the nodular stage, seldom presents with pneumothorax, and it can be cured with a systemic disease treatment, without sequelae. In the other presentation, lung LCH disease typically occurs in adolescents and young adults with an insidious onset. Lung damage develops insidiously, and the disease might not be revealed until the pneumothorax stage [9, 10].

Importantly, PLCH pathogenesis is different between childhood and adulthood, because over 95% of affected adults are cigarette smokers [11]. Thus, PLCH is mainly described in adults with a peak frequency at ages 20–40 years. On the other hand, lung involvement in childhood LCH is less often described, due to much lower occurrence [7, 12–14]. In young adults, computed tomography (CT) scans typically show nodules and cysts, predominantly in the upper and middle lung fields [11, 15]. Adult PLCH histology is characterized by focal CD1a-positive cells, often organized into granulomas, that infiltrate and destroy distal bronchioles, resulting in cyst formation and pulmonary tissue destruction [9]. The evolution of PLCH in young adults ranges from self-regression, upon discontinuing tobacco consumption, to severe respiratory failure [16]. Secondary pulmonary hypertension can shorten life expectancy. Additionally, lung disease can be life-threatening and might require lung or heart-lung transplantation [9, 17].

Exceptionally, within children, even at a young age, LCH may present as severe lung damage, as typically observed in young adults. Management of these exceptional cases of young children with very severe lung disease is challenging; only few case reports have been published [7, 18–27]. Certain described children required management in an intensive care unit (ICU) for respiratory decompensation. Although most patients described remained alive at the publication date, long-term prognosis has remained unclear, and optimal management has not been defined.

Due to recent progress broadening LCH pathogenesis molecular understanding [28], LCH has been associated with a very low mortality rate. Indeed, patients with *BRAF*^V600E^ mutations and MS RO+ LCH historically had the worst prognosis. Currently, these patients typically respond to BRAF inhibitors [29], greatly improving their survival [13].

The present study aimed to provide data on the rare cases of very severe PLCH in childhood to facilitate future treatment strategies development.

## Methods

### Patients and ethics statement

This multicentre, retrospective observational study included patients under 15 years old with PLCH that required at least one ICU hospitalization for respiratory decompensation between 1994 and 2018. All children were registered in French LCH registry [13]. Patients in the registry had to fulfill LCH diagnostic criteria [30]. Additionally their parents had to provide informed consent (Commission Nationale d’Informatique et des Libertés number in France: 909027) for a prospective follow-up. This procedure complied with the Declaration of Helsinki. The registry file was managed by the French reference centre for histiocytosis, located at the Armand Trousseau hospital (Paris, France). Data were recorded prospectively in the registry, starting at patient enrolment. For all eligible patients in the registry files, we collected LCH presentation data, clinical characteristics, outcome, therapeutics, and management during all ICU hospitalizations. When data was missing, we submitted a request for information to the department responsible for the patient.

### Definitions

Disease extension was defined according to international criteria. Single-system (SS) LCH involves only one organ affected. Multisystem (MS) LCH involves two or more organs/systems, and “risk organs” (RO+) included the haematopoietic system, liver and spleen. Lung involvement was defined as a confirmed LCH diagnosis with symptomatic (dyspnoea, cough, cyanosis) or asymptomatic lung disease and characteristic pulmonary findings on a chest X-ray or computed tomography scan. These characteristic findings included symmetric, bilateral reticulonodular opacities, more or less associated with cysts, and a combination of nodules and cysts on a CT scan. Complete pneumothorax was defined as an entire lung collapses on the affected side. Pneumothorax was bilateral if both the right and the left *sides of the lung are concerned.*To quantify dyspnoea, we retrospectively attributed scores, based on the New York Heart Association (NYHA) functional classification: I, no limitation of physical activity; II, slight limitations in physical activity, but ordinary physical activity resulted in fatigue, palpitation, and dyspnoea; III, marked limitations in physical activity, and less than ordinary activity caused fatigue, palpitation, or dyspnoea, but the patient was comfortable at rest; IV, unable to carry on any physical activity without discomfort, and the patient experienced symptoms at rest.

Biopsy date is considered LCH diagnosis date. When a biopsy was available for a molecular study, the *BRAF*^V600E^ mutation was investigated, as previously described [4], and in some cases, other MAPK mutations were investigated. Treatment efficacy was evaluated according to the classification used by the Histiocyte Society [31].

The lung CT score was developed by our group to analyse distribution and extension of nodular and cystic lung lesions and to assess treatment therapeutic impact (Della Valle et al., *under review*). This scoring system evaluated nodules and cysts separately [15]: the chest was divided into 6 fields (upper, middle, and lower fields in the left and right lungs). Each field was scored as follows: 0 = no lesion, 1 = lesions involving up to 25% of the parenchyma, 2 = lesions involving 25–50% of the parenchyma, 3 = lesions involving 50–75% of the parenchyma, and 4 = lesions involving more than 75% of the parenchyma. The maximum score was 24 for extensions in both nodules and cysts.

### Statistical analyses

Statistical analyses were performed with Stata® 13 software. The cut-off date for these analyses was June 30, 2019. Survival analyses included the interval between pulmonary LCH diagnosis and death. In these analyses, patients were censored at the date of the last examination or death. Survival rates were estimated with the Kaplan–Meier method.

## Results

### Demographic and clinical data

**Fig. 1:**
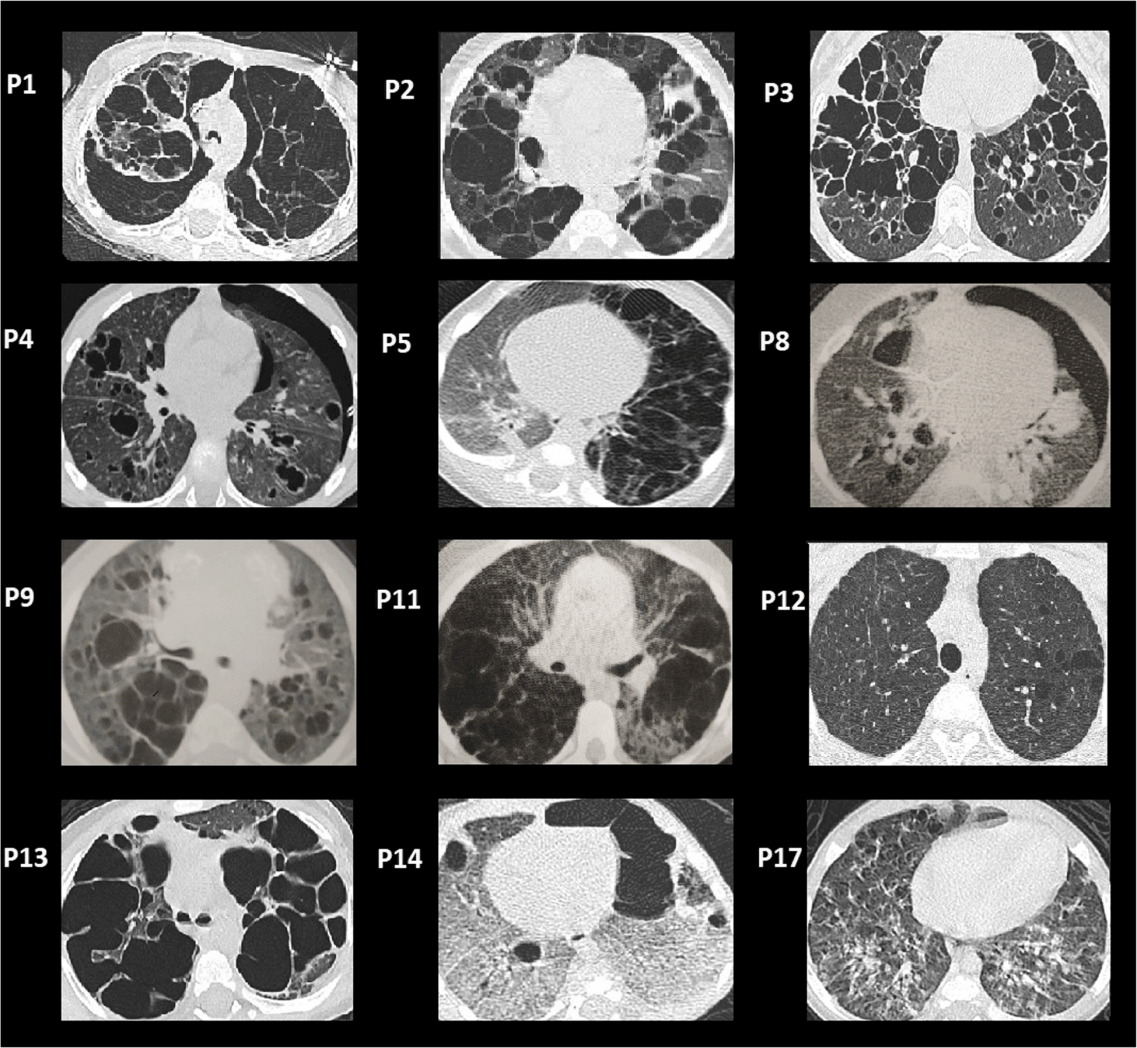
Chest CT scan images (lung window). Initial images of PLCH for 12 of 17 patients. PLCH: Pulmonary Langerhans cell histiocytosis

Among 1482 children under 15 years old that were followed in the French LCH registry between 1994 and 2018, 111 patients (7.4%) had a lung involvement, whereof 17 (1.1%) had severe lung involvement and required hospitalization in an ICU (Table 1). Comparison of PLCH children with and without history of ICU admissions for respiratory failure is showed in Supplemental Table 1. Among the 17 patients, 7 (41%) had RO involvement (liver: *n* = 7; haematologic: *n* = 4; and spleen: *n* = 5) and were classified as MS RO+ LCH; 3 had isolated lung involvement (Lung+ SS LCH); and 7 had Lung+ MS RO-, with other organ involvement (skin: *n* = 5; bone: *n* = 2; pituitary: *n* = 2; lymph node: *n* = 2; tumorous central nervous system: *n* = 1). The median age of all 17 patients was 1.2 years (range, 2 days to 13.7 years) at the initial LCH diagnosis. The median ages at the LCH diagnosis were 0.7 years for patients with MS RO+, 1.2 years for patients with Lung+ MS RO- with other organ involvement; and 5.0 years for patients with Lung+ SS. Lung involvement was diagnosed at the initial LCH diagnosis in 14 patients, and 3 children developed lung involvement at LCH reactivation. The latter 3 patients had an initial normal chest X-ray at diagnosis; the lung involvement was diagnosed at a median delay of 2.4 years (range, 0.6 to 2.4) after the initial LCH diagnosis. At the PLCH diagnosis, 3 patients had no respiratory symptoms, 14 had dyspnoea (NYHA dyspnoea score II: *n* = 2; NYHA dyspnoea score IV: *n* = 12), and 11 had pneumothorax. The available CT scans acquired at PLCH diagnosis are shown in Fig. 1. LCH biopsy samples were available for 11 patients. A molecular study showed that 4 patients had the *BRAF*^V600E^ mutation, and one patient had a *MAP2K1*^F53_Q58del^ mutation.

**Table 1:** Characteristics of 17 children with LCH with severe lung involvement

Characteristics	*n* = 17 (% of cases)
Male	8 (47)
Female	9 (53)
Age at diagnosis (median)	1.2 y
< 3 years	12 (71)
> 3 years	5 (29)
Lung+ SS LCH	3 (18)
Lung MS RO- LCH	7 (41)
Lung MS RO+ LCH	7 (41)
Bone involvement	6 (35)
Skin involvement	11 (65)
Pituitary involvement	4 (24)
Tumorous central nervous system	2 (12)
Liver involvement	7 (41)
- including sclerosing cholangitis	1
Hematologic involvement	4 (27)
Spleen involvement	5 (29)
Lung involvement	17 (100)
Lymph node involvement	6 (35)
First line VLB–steroid regimen	16 (94)
Second-line therapy	12 (71)
2 CDA	7 (41)
VP-16 Ara-C	3 (18)
Targeted therapy	2 (12)
LCH reactivation	8 (47)
*BRAF*^V600E^ mutation (mutated/investigated cases)	4/11
MAP2K1 mutation	1

*Abbreviations: DAS* Disease Activity Score, *LCH* Langerhans cell histiocytosis, *PLCH* Pulmonary LCH, *VLB* vinblastine, *VP16 Ara-C* Etoposide-Cytarabine, *2CDA* Cladribine, *RO* Risk organs involved, *MS* Multi-System, *SS* Single-System

### Characteristics of ICU hospitalization

**Fig. 2:**
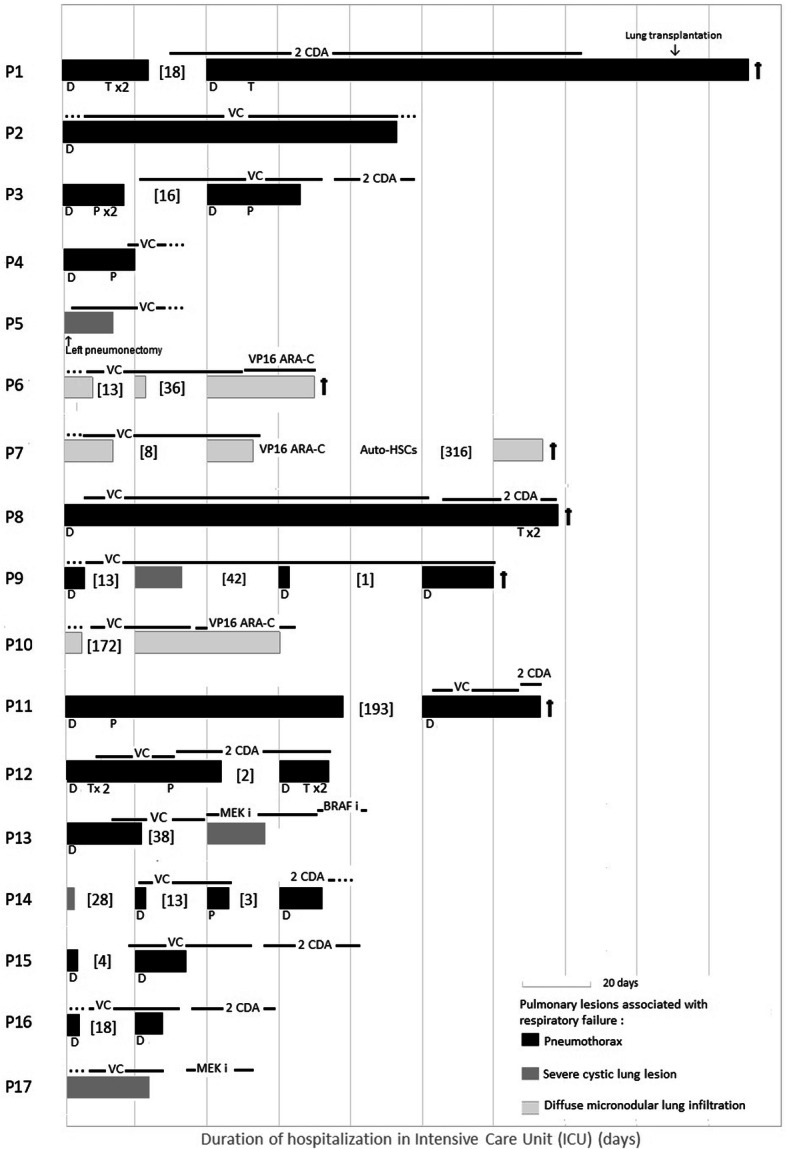
Intensive care unit (ICU) hospitalizations and management for 17 patients with pulmonary Langerhans cell histiocytosis. Solid bars represent one ICU stay, and the length of stay is proportional to the scale bar (each section on the X-axis = 20 days). Bracketed numbers are the number of days between ICU stays. □ indicates death. Pulmonary lesions associated with respiratory failure are indicated with shading (black: pneumothorax; dark grey: severe cystic lung lesion without pneumothorax; light grey: diffuse micronodular lung infiltration). Anti-LCH treatments are specified above each ICU stay, and the treatment time is indicated with lines (VC: vinblastine corticosteroid; VP16 Ara-C: Etoposide-Cytarabine; 2CDA: Cladribine; Mek i: MEK inhibitor; BRAF i: BRAF inhibitor). Points before or following the lines indicate ongoing treatments for unspecified times. Pneumothorax treatments are indicated under each ICU stay (D: drainage, T: talcage, P: pleurectomy by thoracotomy)

A total of 35 ICU hospitalizations were analysed, representing a median of 2 ICU hospitalizations per patient (range: 1 to 4, Fig. 2). The median time between the PLCH diagnosis and the first ICU admission was 9 days. The median ICU stay was 14 days (range: 2 to 151). For patients that required multiple ICU hospitalizations (*n* = 12), the median interval between ICU hospitalizations was 17 days (range: 1 to 316 days).

Among the respiratory decompensations that led to ICU hospitalizations, 12 children experienced a complete pneumothorax (representing 22/35 (63%) reported ICU hospitalizations). Among these, 5 patients experienced a bilateral pneumothorax. All 22 pneumothorax events were managed with chest tube drainage; 9/12 patients (75%) also received pleurodesis (including thoracotomy pleurectomy surgery: *n* = 4; talc or chemical pleurodesis: *n* = 4; or a combined method: *n* = 1). Only 2 children did not experience a pneumothorax relapse after pleurodesis; the other 7 children experienced pneumothorax recurrences and required several pleurodesis procedures (median: 2 pleurodeses per patient, range: 1 to 5).

In 8/35 (23%) ICU hospitalizations, the respiratory distress syndrome was associated with a diffuse micronodular lung infiltration in the context of MS LCH.

Finally, in 5/35 (14%) ICU hospitalizations, the respiratory distress syndrome was associated with a severe cystic lung lesion, and among these, one patient underwent a left complete pneumonectomy 24 days after the LCH diagnosis.

In 9/35 (26%) ICU hospitalizations, a viral respiratory infection (VRI) was identified at admission. These VRIs included respiratory syncytial virus (RSV), rhinovirus, human metapneumovirus, influenza A virus, and parainfluenza virus 3.

### Management during ICU hospitalization

All patients required oxygen therapy during ICU hospitalization. Additionally, 10 patients (59%) required invasive mechanical ventilation (IMV), and the median cumulative duration was 17.5 days per patient. One patient required a tracheotomy, due to prolonged IMV (48 days of IMV) without the possibility of extubation. One patient acquired an IMV-associated nosocomial pneumonia. In 3 ICU hospitalizations (diffuse micronodular lung infiltration: *n* = 1; pneumothorax: *n* = 2), non-invasive ventilation was administered without IMV.

The first-line LCH-specific therapies (Table 2) included the combined vinblastine corticosteroid (VC) therapy for 16 patients and 2-Chlorodeoxyadenosine (2CDA) therapy for one patient. Among the 16 patients that received VC, only 3 cases showed evidence of a response and remained alive with no LCH reactivation. The patient that received 2CDA did not respond; therefore, a lung transplantation was undertaken, and the patient died, following a solid organ transplant-associated acute graft-versus-host disease, as previously reported by Galambrun et al. [24] Among the 13 non-responders to VC, one died after VC therapy without evidence of any response.

**Table 2:** Characteristics of PLCH and therapies at diagnosis and during follow-up

Characteristic	At PLCH diagnosis *n* = 17	After the last ICU hospitalization *n* = 11^a^	At last visit *n* = 11^a^
Median interval after the PLCH diagnosis	_	63 days (range: 4–966)	11.2 years (range: 0.5–16)
Respiratory symptoms			
No symptoms	3 (20)	2 (25)	9 (82)
NYHA 2	2 (13)	5 (38)	2 (18)
NYHA 3			
NYHA 4	12 (71)	4 (36)	
Oxygen requirement	12 (71)	4 (36)	0
LCH therapy			
VLB Steroid	16 (94)	3 (27)	
VP16 ARA-C		1 (9)	
2CDA	1 (7)	5 (45)	
Targeted therapy		2 (18)	2 (18)
DAS Score, Median [min, max]	5 [3,23]	3 [1, 3]	1 [1,1]
CT Scores,	(11 evaluated cases)	(7 evaluated cases)	(7 cases evaluated)
Cyst, median [min, max]	12 [0,24]	10 [0,21]	10 [0,18]
Nodule, median [min, max]	0 [0,6]	0 [0,3]	0 [0,2]

Values are the number of patients (%), unless indicated otherwise

—: not applicable

*Abbreviations: DAS* Disease Activity Score, *CT* computed tomography, *ICU* intensive care unit, *LCH* Langerhans cell histiocytosis, *NYHA* New York Heart Association, *PLCH* Pulmonary LCH, *VLB* vinblastine, *VP16 Ara-C* Etoposide-Cytarabine, *2CDA* Cladribine

^a^6 patients died in the last ICU

**Fig. 3:**
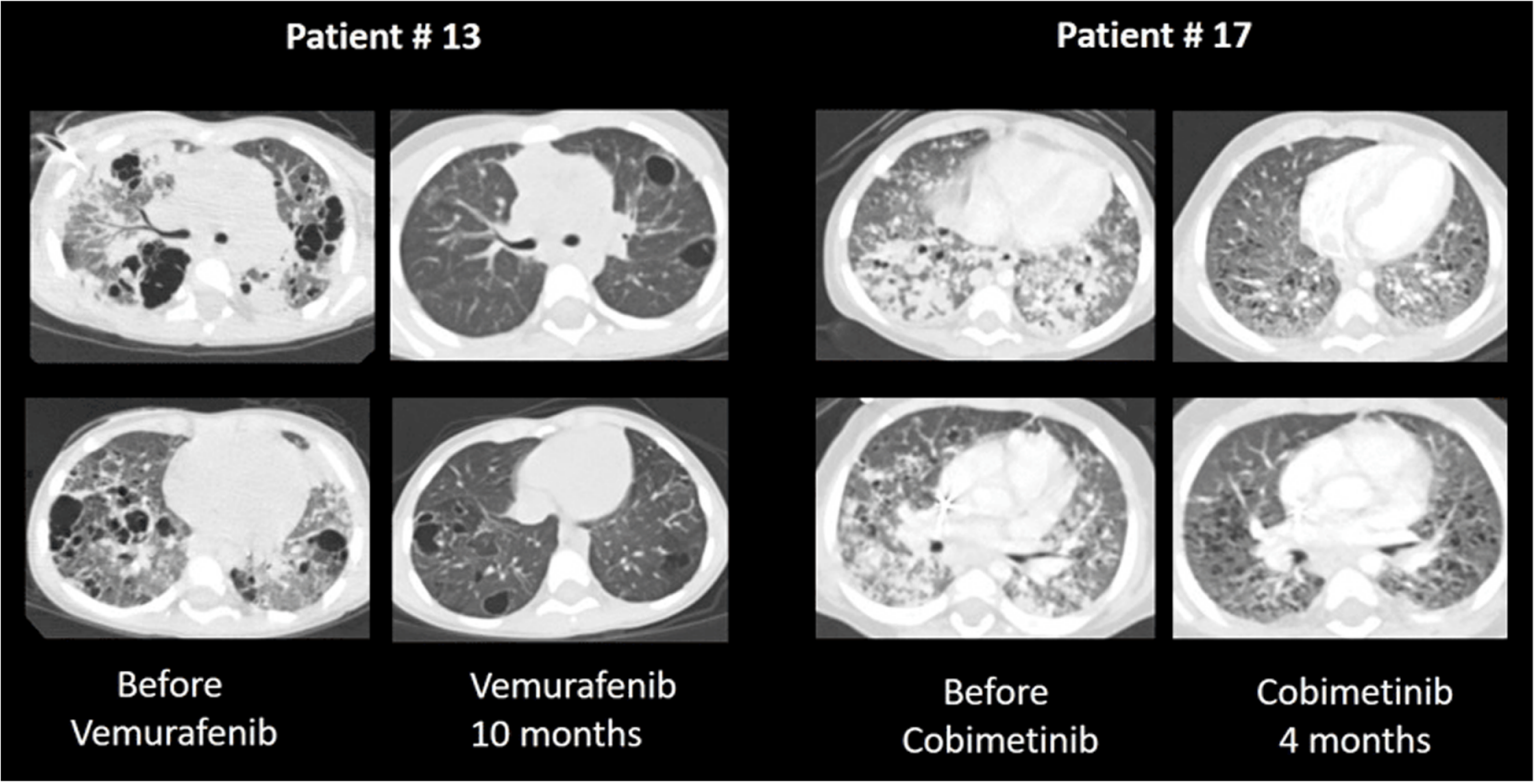
Chest CT scan images (lung window) before and after initiation of *target therapy.* Images show lungs before and after the initiation of a BRAF-kinase inhibitor (Vemurafenib) and a MEK inhibitor (Cobimetinib)

Seven patients were treated with 2CDA as a 2nd line therapy. Of these, 5 responded without LCH reactivation, and 2 did not respond and died. Three patients received a combination of etoposide and cytarabine. Of these, 2 patients did not respond and subsequently died, and one showed a partial response (2 reactivations). Finally, 2 patients were treated with a targeted therapy (BRAF inhibitor: *n* = 1; MEK inhibitor: *n* = 1). Both showed responses without reactivation at a median of 6 months after ICU admission (Fig. 3).

### Outcome

**Fig. 4:**
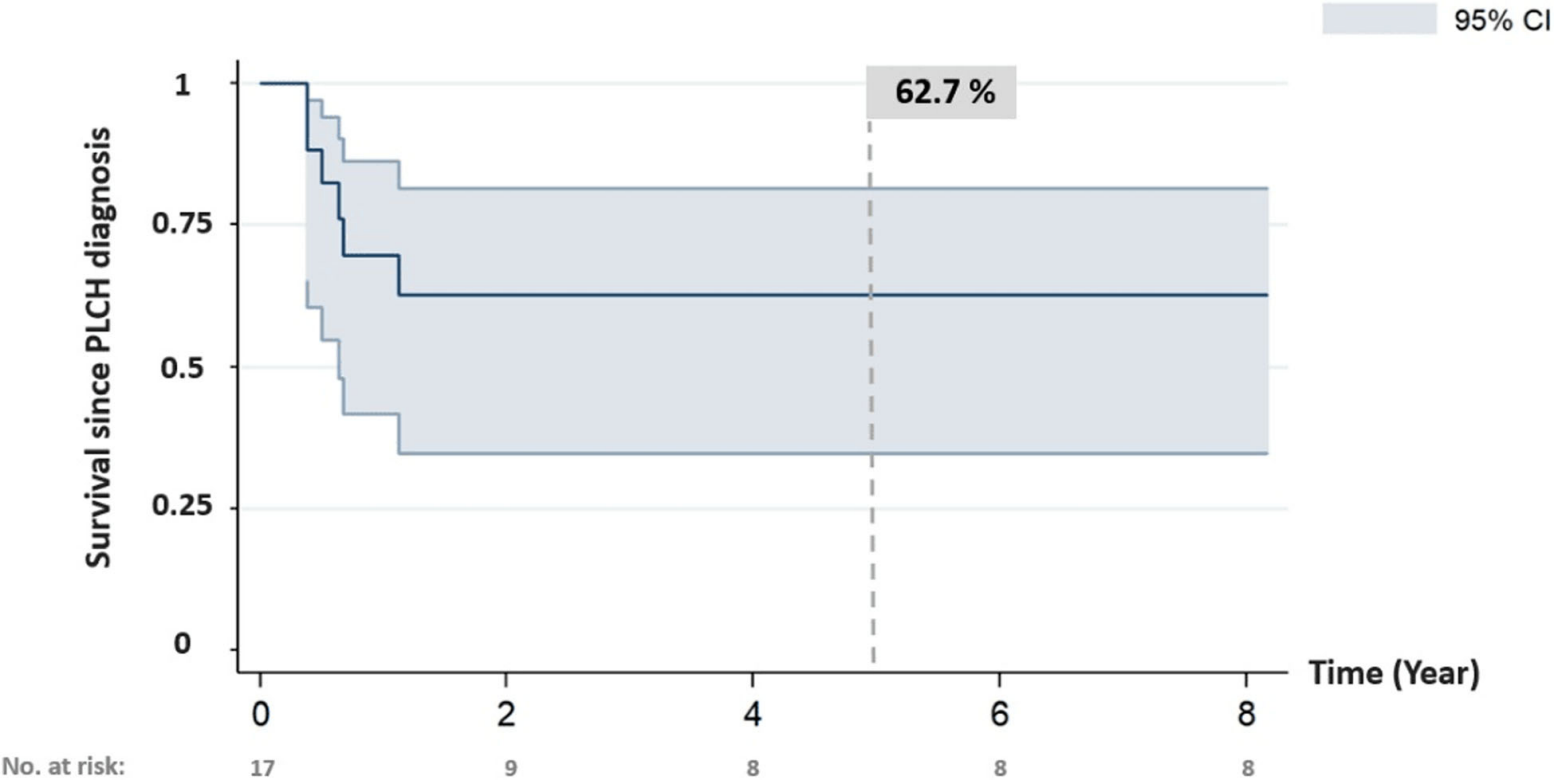
Overall survival after pulmonary Langerhans cell histiocytosis diagnosis for patients with severe lung involvement (*n* = 17)

A total of 6 children (35%) died. Median time to death after PLCH diagnosis was 6.9 months (range: 4.6 to 13.7 months). Estimated 5-year survival rate after PLCH diagnosis was 62.7% (95%CI: 34.8–81.3%; Fig. 4). Five patients died from respiratory failure during the ICU hospitalization (following repeated pneumothoraxes: *n* = 3; diffuse micronodular lung infiltration in the context of MS: *n* = 2) and one died from graft-versus-host disease after lung transplantation (Table 3) [24]. For 4/6 children with a determined lung CT score, the median radiological score at the PLCH diagnosis was 16 for cysts and 0 for nodules but this score was not available for the 2 patients with diffuse micronodular lung infiltration in the context of MS disease.

**Table 3:** Characteristics of children that died of PLCH and comparison with surviving patients

Characteristics	Deceased patients, ***n*** = 6	Surviving patients, ***n*** = 11	***P*** value
Age at PLCH diagnosis, median [min, max]	1.0 y [0.2–5.0]	1.4 y [0.1–13.7]	0.8
RO- LCH disease	3	7	0.6
RO+ LCH disease	3	4	
Maximal DAS Score, median [min, max]	13.5 [7,27]	5 [4,10]	0.04
First-line LCH therapy			
VLB Steroid	5	11	0.4
2CDA	1	0	
Second-line LCH therapy			
2CDA	2	5	0.4
VP16 ARA-C	2	1	
Targeted therapy	0	2	
CT Score at PLCH diagnosis	(n = 4 evaluated cases)	(n = 9 evaluated cases)	
Cyst, median [min, max]	16 [8,24]	10 [1,18]	0.14
Nodule, median [min, max]	0 [0,0]	3 [0,9]	0.08
*BRAF*^V600E^ mutated LCH	0/2 investigated cases	4/9 investigated cases	0.5
Time to death after PLCH diagnosis, median [min, max]	6.9 months [4.6,13.7]		
Cause of death			
Repeated pneumothorax	3	_	
Diffuse micronodular lung infiltration	2		
Post lung transplant	1		

Values are the number of patients, unless indicated otherwise

*Abbreviations: DAS* Disease Activity Score, *CT* computed tomography, *LCH* Langerhans cell histiocytosis, *PLCH* Pulmonary LCH, *RO* Risk organs involved (+) or not involved (−), *VLB* vinblastine, *VP16 Ara-C* Etoposide-Cytarabine, *2CDA* Cladribine

The 11 surviving children had a median follow-up of 11.2 years after the last ICU (range: 0.4 to 15.7 years). Three children required prolonged supplemental oxygen after the ICU for a 1 month to 2.5 years period, but none remained under oxygen at last contact. Nine survivors (including one case that underwent a left complete pneumonectomy) were leading a normal life and practicing sports, despite imperfect lung function and radiological sequelae. Only 2 patients remained symptomatic, with NYHA II dyspnoea. Seven of the surviving patients had an imaging follow-up available. At the PLCH diagnosis, 7/11 patients with a determined lung CT score had median radiological scores of 12 for cysts and 2 for nodules. At the last visit, these patients had median scores at 10 for cysts and 0 for nodules.

## Discussion

In this study, we described a cohort of 17 children with severe lung LCH registered in the last 30 years in the French LCH registry. This registry was based on the nationwide multicentric inclusion of patients treated within a long-standing, well-structured network of oncopaediatric and pathologist practices [4, 13, 32]. Consequently, it is likely that nearly all children in France presenting LCH during the study period were reported in this registry. Accordingly, the presentation of severe lung LCH could be considered very rare; it represented 1% of all childhood LCH cases and ~ 10% of all LCH cases with lung involvement in children [4, 32]. In the literature, pulmonary involvement in children with LCH has been reported in many studies, but severe cases have been very rarely described [5, 7, 14]. For example, Ha et al. described 24 children with PLCH, but only 2 had respiratory complications [6]. To our knowledge, this study was the first to focus on severe childhood PLCH with a long follow-up (median follow-up for survivors: 11.2 years after ICU). Most of our knowledge about PLCH is mainly based on adult pulmonology studies, because lung LCH involvement mostly affects young adults [9, 17]. Several studies have shown that tobacco consumption directly affected adult PLCH; however, we lack explanations for the onset of PLCH in young children. Passive smoke exposure has not been identified as a risk factor for PLCH in children.

In this study, the children with severe lung disease were very young (median age at LCH diagnosis: 1.2 years), as previously reported [14, 33]. All but one displayed severe lung damage at the time of the LCH diagnosis. Most damage was represented by extended and probably insidious cystic lesions. The main cause of ICU hospitalizations was pneumothorax, but we also observed respiratory failure associated with severe cystic lung lesions (without pneumothorax) and diffuse micronodular lung infiltrations. Of note, 26% of ICU hospitalizations were associated with an identified VRI, probably representing a common cause of respiratory decompensation. Accordingly, we strongly recommend annual vaccinations against influenza. Moreover, RSV prophylaxis with palivizumab should be considered during the first year after a PLCH diagnosis, as previously proposed for other severe lung disorders in young children [34].

The 5-year survival rate was 62.7%. This low survival rate testified to the severity of this rare LCH presentation, particularly during the critical period of the first year after PCLH diagnosis. Indeed, all deceased patients died within a median of 7 months (range: 4.6–13.7) after the PLCH diagnosis. For hospitalizations with pneumothorax, recurrences of pneumothorax were common, despite pleurodesis [19, 20, 25, 26]. Optimal management for preventing pneumothorax recurrences has not been clearly established for children with LCH. For adults, surgical pleurodesis might be indicated in cases of recurrence, but a pleurectomy is typically avoided for patients that might be candidates for lung transplantation [17]. However, recurrent pneumothorax, despite pleurodesis, remains an issue, and further studies are needed to determine the best management for reducing the risk of pneumothorax recurrence in PLCH [11, 35, 36]. A pneumonectomy might be considered when the damage is localized, as described for one patient in this study.

Our investigation of therapeutic efficacy in controlling PLCH showed that a minority of patients responded to VC (3/16), and 5 of 8 patients treated with 2CDA responded without reactivation. Conversely, none of the 3 patients treated with the etoposide-cytarabine combination (used in the 90s, but no longer recommended) survived without reactivation. Despite the low number of patients treated in each group, our findings highlighted the difficulty in managing LCH disease with severe lung involvement. In adults, VC is not indicated for treating PLCH, due to the poor response rate, and 2CDA is indicated as the first-line therapy for PLCH, when systemic chemotherapy is required [37]. For children, VC therapy is suitable as a first-line treatment for all LCH involvements, including PLCH [30]. However, in our study, the VC response rate was low for children with severe PLCH. 2CDA is an alternative option, but it might have adverse effects, such as a prolonged immunosuppression. Due to the small number of patients included in this study, it was difficult to draw conclusions about the optimal chemotherapy for treating PLCH. Notably, targeted therapies might also represent an interesting option. Two of our patients were treated with targeted therapy and showed continued clinical and radiological improvement, from the beginning of therapy, and both remain alive currently.

Molecular knowledge of histiocytosis has provided new avenues of potential progress in histiocytosis, particularly in terms of treatment [28]. In patients with LCH associated with *BRAF*^V600E^ mutations (55% of children with LCH [4]), a BRAF inhibitor showed efficient effects. The other mutations associated with LCH (*MAP2K1*, *BRAF*indel) were responsive to MEK inhibitors [28]. Thus, considering the severity of this LCH presentation, we believe that the use of targeted therapy would be justified, because it showed rapid efficacy in LCH [29, 38, 39].

Our findings also justified the use of an adapted initial intensive management as our survivors experienced long-term, good quality of life and improved lung function, despite persistent radiological lung lesions (chronic cysts). No survivors developed late respiratory failure that required a lung transplantation [40].

## Conclusions

We showed that, although the lung is no longer considered an RO in childhood LCH, some severe lung presentations were associated with an adverse prognosis. These presentations represented a very tiny fraction of all lung involvement in LCH, but their management remains highly challenging. Future studies should define optimal management approaches for pneumothorax complications and infectious prophylaxis, particularly during the first year after the PLCH diagnosis. Moreover, to optimize LCH therapy for these patients, future studies should evaluate using targeted therapies early in the development of severe PLCH in young children.

## Supplementary information

**Additional file 1: Supplemental Table 1.** Comparison of PLCH children with (*n* = 17) and without (*n* = 94) history of ICU admissions for respiratory failure. Data from the whole LCH cohort of children bellow 15 years of age at diagnosis (*n* = 1482) is also reported in order to give reference for the reader.

## Data Availability

The datasets used and/or analysed during the current study are available from the corresponding author on reasonable request.

## Abbreviations

2CDA: 2-Chlorodeoxyadenosine
CT: Computed Tomography scan
DAS: Disease Activity Score
ICU: Intensive Care Unit
IMV: Invasive Mechanical Ventilation
LCH: Langerhans cell histiocytosis
Lung+ MS RO+ LCH: lung involvement with Risk Organ
Lung+ MS RO- LCH: lung involvement with other organ
Lung+ SS LCH: Isolated lung involvement
MS: Multi-system
NYHA: New York Heart Association
PLCH: Pulmonary LCH
RO +: Risk Organs involved
SS: Single-System
VC: Vinblastine Corticosteroid
VLB: vinblastine
VP16 Ara-C: Etoposide-Cytarabine
VRI: Viral Respiratory Infection
